# Political theories of the business corporation

**DOI:** 10.1111/phc3.12892

**Published:** 2022-12-17

**Authors:** Rutger Claassen

**Affiliations:** ^1^ Department of Philosophy and Religious Studies Utrecht University Utrecht The Netherlands

## Abstract

Business corporations are important, often powerful actors within the economy. They are able to exercise power over other actors, such as employees, consumers and nation‐states. This contribution discusses how corporate power is constituted (ontological question), for what purpose it should be exercised, (normative question) and how it should be controlled (governance question). It focuses on the competing anwers to these questions that have been proposed by three political theories of the corporation. Concession theories emphasize the state's role in chartering corporations, and hence require corporations to act in the public interest. Contractualist theories present corporations as tools for contract partners (most often shareholders), with corporate purpose focused on the benefits to these partners. Real entity theories focus on the corporation as an autonomous, separate entity with a purpose to be determined by the group seeking incorporation.

## 1 INTRODUCTION

Business corporations make decisions with respect to prices, working conditions, location, tax strategy, etc. These decisions often have a substantial impact on consumers, citizens, employees, shareholders and others. Corporations regularly exercise immense economic, political and cultural power.^1^ Corporate power has been a recurring source of public concern, e.g. with the role of the banks during the financial crisis, the Big Tech companies' influence on democratic decision‐making, and the energy companies' role in the climate crisis. In political philosophy, political (as opposed to purely moral) theories of the business corporation have gained ascendency.^2^

Business corporations are a species of corporations. Corporations are characterized by having a separate legal personality. With legal personality comes the ability to own property and make contracts in the corporation's own name, a centralized management structure representing the corporation, and the right to impose rules on the corporation's members (Ciepley, 2013, p. 141). Corporations arose as Medieval jurists rediscovered the Roman legal concept of the *universitas*. It was gradually understood as an abstract legal person (‘persona ficta’), i.e. not a person of flesh and blood (natural person), that can assume some of the roles and responsibilities of natural persons. In the Middle Ages, corporate status was given to such diverse entities as towns, monasteries, universities and guilds. In the 17^th^ century, governments started to grant concessions to commercial entities for the purpose of overseas trading, i.e. the Dutch and English East India Companies.^3^ Later, this was extended to other specific tasks, such as the construction of canals and bridges, banking and insurance. From the 19^th^ century onwards, corporate status was granted for all business purposes.

In addition to legal personality, business corporations have five specific legal characteristics, in most jurisdictions.^4^ The first three relate to the capital structure. (i) Shareholders cannot withdraw their capital from the corporation (asset lock‐in). Instead, they receive shares, with the option of a reward (dividend). This enables corporations to invest in long‐term strategies. (ii) Creditors of the corporation have no claim to the private assets of shareholders. This limits shareholders' liability to the size of their investment (limited liability). (iii) Conversely, creditors of shareholders have no claim to the assets of the corporation (entity‐shielding). Shareholders' investment is thereby protected against the private debts of other shareholders. Together these three characteristics create a “wall” between shareholders and the corporation, allowing the corporate assets to form a separate pool of wealth. This legal entity, represented by its board, could settle on a course of action antithetical to the interests of its shareholders. To prevent this, (iv) shareholders have the right to elect the board of directors, and thus have indirect, but ultimate, control (v) Finally, while shareholders cannot withdraw their shares, they can sell them. For the public corporation this takes place on a stock exchange; for the closed corporation the transaction is private. Shareholders thus regain flexibility regarding their assets, and they can put pressure on boards with the threat of sale (the “market for corporate control”).

Uncoincidentally, larger and more powerful firms are almost always business corporations.^5^ The legal features of the corporate structure help to explain their ability to grow large, since they enable the pooling of large amounts of capital under one centralized command structure.^6^ In thinking about corporate power from the point of view of political theory, we can usefully distinguish three questions. First, how is such power constituted? (*ontological question*). Second, for what purpose should corporate power be exercised? (*normative question*). Third, how should corporate power be controlled? (*governance question*).

Answers to the ontological question give us an interpretation of business corporations' *legal personality*. Traditionally, three theories provide competing answers: the concession theory, contractualist theory and real entity theory.^7^ The normative and governance questions both speak to the issue of *legitimacy*. Corporate power is legitimate to the extent that it is exercised in line with a purpose that society accepts as legitimate, and the allocation of control is recognized as legitimate. The three theories – based on their ontology – propose answers as to what kind of corporate purpose can be legitimate. In Sections 2, 3 and 4 I discuss each of the three theories in turn, taking together the ontological and the normative question. In Section 5 I provide a separate discussion of the governance question, since the three theories are more loosely aligned with answers as to what corporate governance structure is warranted to keep corporate decision‐making in line with its purpose.

## 2 CONCESSION THEORY

Concession theories emphasize the role of the state in granting legal personality to groups wanting to incorporate. Corporations are artifacts of law. Stressing this artificial origin, the theory is also referred to as ‘artificial entity theory’. Normatively, the purpose for which corporations must act is, at least partly, a public purpose, and the state has a legitimate interest in closely regulating them.^8^

Let's start with the ontological question. Corporations' constitution of their legal personality was originally related to a specific grant. In Roman law, a concession from the state was required to start a *universitas* (Avi‐Yonah, 2005, p. 774). In a charter of incorporation, the rights and obligations of the legal person toward the state were laid down. The general understanding was that a corporation had to fulfill a public interest. In the early modern period most economic activity did not take place in corporations, but in sole proprietorships or partnerships (Avi‐Yonah, 2005, p. 784). Due to the state's selectivity in granting concessions, business corporations were often monopolists. This drew widespread criticism. Adam Smith, for example, argued that such monopolies were a covert tax on other citizens (Smith, 1994, p. 814). In the 19^th^ century, a watershed occurred, when most countries passed general incorporation laws. From the 19^th^ century onwards, access to the corporation as a legal form was ‘privatized’. The prior assessment of whether a new corporation served a public interest was dropped; incorporation became a right subject only to administrative formalities. Immediately, the number of corporations rose dramatically, given the clear benefits of the corporate structure to investors.

Concession theories were the dominant theories up until the privatization of the corporation.^9^ A concession is an exception to the normal rules, selectively handed out by the state to a limited group of people for a concrete public interest. The shift to a general incorporation regime appeared to many critics to invalidate the ontological thesis of corporations' artificial creation by law. When creating a new corporation is open to anybody, there is no such concession. However, concession theory has recently reappeared in political theory and legal scholarship, probably inspired by renewed concerns about corporate power.^10^ Even though anyone can incorporate, corporations still have a concession in the sense of a legally privileged position compared to others with whom they interact, like workers or consumers. And states remain indispensable for granting these privileges, for (some of) these legal features cannot be created through private contracting (Ciepley, 2013, pp. 144–45).

This brings us to the normative question. Being a gift from the state, corporations' legal features are interpreted as ‘privileges’, which trigger a demand of reciprocity. As ‘franchise governments’ (Ciepley, 2013, p. 151), they must serve a public interest, as determined by the state through the charter of incorporation. Concession theory doesn't deny that the incorporators have their private purposes with the enterprise, but merely demands that this private mission (also) fulfills a public interest. Historically, trade (guilds), spiritual salvation (churches), education (universities) etc. were deemed to be *also* in the public interest. Can such a normative position survive, in our era of general incorporation laws where corporate purpose seems to be fully privatized (Avi‐Yonah, 2005, p. 792; Singer, 2018a, p. 172)?

Today one can accept that the legal features of business corporations are (necessarily) state‐created, but also hold that this is merely an off‐the‐shelf legal framework states make available to facilitate purely private purposes.^11^ In this spirit, critics argue that concession theory shows too little respect for entrepreneurial freedom. In a modern society, businesses should not be seen as an extension of the state, any more than other private collective agents, such as sport clubs, churches or universities (Orts, 2013, p. 21).

However, concession theorists think the theory has not lost relevance in the post‐privatization era. The requirement of ‘public interest’ needs to be interpreted more broadly than the fulfillment of a concrete public task. Now that anyone can start an incorporated business, entrepreneurs are legally allowed to take their private self‐interest as their personal objective. Nevertheless, there is still is a public good behind this, that is, the creation of prosperity in general. As David Ciepley argues: ‘Business incorporation too is a state program. It is a state program for economic growth’ (Ciepley, 2019a, p. 1004). Corporations operate in the public interest, when through the invisible‐hand mechanism of the market they create economic growth. The weight of the argument is now not on governmental provenance per se. For states create many other legal institutions (e.g. property law) which are also meant to enable and incentivize private individuals to create wealth, without demanding a favour in return.^12^ Corporate legitimacy comes to rest more on the conformity of corporate practices with the public interest, broadly understood as success in economic development (Claassen, 2021).

This raises further questions how exactly to understand this public interest. Concession theories often argue that the purpose shouldn't be narrowly focused on the financial gain of shareholders only; the public interest also includes the legitimate interests of third parties and implies a balancing act of all these interests, which adds up to ‘the public interest’ (see further 5.1 and 5.2).

## 3 CONTRACTUALIST THEORY

Contractualist theory holds that ontologically corporations should be understood as arising from contracts between contracting partners. The theory is also called ‘aggregate theory’, expressing that corporations are to be seen as aggregations of their participants. Normatively, the purpose of business corporations is to benefit the interests of these contracting partners (or members). As for non‐business corporations, these could be any set of partners starting a corporation. For business corporations, the relevant contracting partners have nearly always been taken to be the shareholders, for reasons explained in this section. However, this is not a matter of logical necessity, a point to which we return later (see 5.2).

Contractualist theories enjoyed increased popularity in the second half of the 19^th^ century, to explain the rise of business corporations after free incorporation was established. Its appeal owed a lot to the apparent continuity between corporations and partnerships. In Roman law, the *societas* provided the model for partnerships, in which members directed the common entity for the benefit of their own interests. In the 19^th^ century, the partnership still seemed to provide the most appropriate mental map to interpret freely incorporated new businesses. While the former partners would no longer (co‐)own their partnership, they would now own shares in the corporation. They continued to reap the benefits of any profits, and also often remained in the position of authority, albeit now as board members of a separate entity. However, this analogy with partnerships disregards important aspects of the separate legal existence of the corporate person. The shareholders are no longer, like partners, the corporations' owners. Corporate assets are owned by the legal person itself, shareholders merely own their shares (Ireland, 2018, pp. 300–306).^13^ Shares do not entitle them to dividends, this depends on whether boards judge payments feasible and appropriate. Also, partners are fully liable for downside risks, while limited liability partly shields shareholders from these risks. Only the corporation itself is liable to its creditors (Harris, 2006, p. 1470; Mark, 1987, p. 1459).

Since the 1970s, contractualism was revived in the guise of agency theory (Jensen, 2002; Jensen & Meckling, 1976). Agency theory provides contractualism with economic foundations. Corporations are now understood not as emerging from contracts *between* shareholders, but as a ‘nexus of contracts’ of all parties involved (shareholders and other stakeholders) *with* the corporation. With this reformulation, contractualism now accommodates the separateness of the shareholder position from the corporate legal entity. The latter is ‘the common counter‐party in numerous contracts with suppliers, employees, and customers, coordinating the actions of these multiple persons through exercise of its contractual rights’ (Kraakman et al., 2017, p. 5).

Agency theory's explanatory contribution lies in analyzing agency problems between shareholders and boards/managers (see Section 5.2). When corporate law and practice is focused on a reduction of agency costs, this would lead to economically optimal outcomes. On a normative level, this provides a defense of the focus on shareholder value as the relevant corporate purpose. All parties involved can secure their interests via voluntary contracting, hence achieve maximally feasible well‐being, except one: the shareholders. Their primary interests—dividend payments and rising share prices—are not contractually guaranteed. Profits are uncertain, and even if there any, the board may decide to reinvest them. Shareholders are therefore the only party in a vulnerable position, as ‘residual claimants’. To ensure that shareholders will have an incentive to invest in a structure which puts them in such a vulnerable position, agency‐inspired corporate law assigns a fiduciary duty of boards towards them (in contrast to the merely contractual duties to all others). The corporation is to be run for the purpose of its shareholders' interests.

Through this analysis, agency theory explains why shareholder value maximization should be a corporation's purpose. However, this normative focus on shareholder value is not a defense of shareholder egoism. True, when agency problems can be overcome, shareholder value is maximized. But when shareholder value is maximized, agency theory claims, this maximizes general social welfare as well. Hence, while the agency‐based version of contractualist theory originates in economics, it can also be interpreted as a *political* theory. The focus on shareholder value is not an end‐in‐itself, but an instrument towards this societal purpose (Kraakman et al., 2017, pp. 28–29). This was popularized by Milton Friedman's famous statement that the social responsibility of corporations is to increase their profits (Friedman, 1970). Agency theory became widely spread in business schools, transformed into shareholder‐oriented laws, and embraced by judges, so much that at the end of the 1990s scholars declared the ‘end of history’ for corporate law to have arrived (Hansmann & Kraakman, 2001).

There is one important caveat: there should be no externalities, i.e. interests of third parties that cannot be adequately protected by contract. Or, if there are, government should adequately protect these interests by imposing rules that internalize them (Jensen, 2002, p. 239). The corporation's own purpose must remain unburdened by explicit public objectives. Agency‐based contractualism hence relies for its plausibility on a sharp division between private and public spheres. Critics of shareholder primacy argue that in a world in which ‘external costs’ are ubiquitous, and governments are often incapable of regulating, such a neat division becomes untenable.^14^ This has led to a lively debate (see also 5.2), with defenders of shareholder primacy standing their ground (Bebchuk & Tallarita, 2020).

## 4 REAL ENTITY THEORY

Real entity theory presents corporations as independent entities, emphasizing their separate existence from the interests of a chartering state or investing shareholders. Ontologically, law's recognition through incorporation is a mere response to the existence of a real group personality. The group will is expressed as the corporate purpose in the charter. Normatively, corporations should focus on realizing this purpose.

Real entity theory was introduced by Otto Gierke (in Germany) and Frederik Maitland (in the UK) at the end of the 19^th^ century (Gierke, 2014; Maitland, 2003). Like concession theory, real entity theory sees the corporate person as a separate legal entity, which cannot be reduced to the will and interests of the aggregate of its members (as in contractualism). Both theories also agree that law must recognize this entity for the corporation to participate in legal transactions in its own name. However, real entity theory interprets the state's chartering as a recognition in law of a pre‐legal group personality. Otto Gierke looked at the medieval German tradition in which society was best characterized as a ‘community of communities’, with guilds, monastic orders, cities, and universities often able to operate with a measure of internal autonomy. Approval of and supervision by an authorizing state was either absent or seen as merely confirming the internal life of the group.^15^

Real entity theory became a serviceable template for interpreting the large business corporations that came to dominate the economy in the first half of the 20^th^ century. In them shareholders no longer were also the directors or managers. A separate class of managers came to dominate decision‐making and shares came to be dispersed over many anonymous investors. The is the famous ‘separation of ownership and control’ (Berle & Means, 2009). With the state having privatized control, and shareholders dispersed, the board assumes central importance, as representing the will of the corporation as a separate entity.^16^ Real entity theory theoretically articulates this historical shift. It points to the board as having a fiduciary duty not towards a particular group, but towards the corporation itself. The corporation receives its purpose from the charter and is therefore bound – indirectly – to the expressed will of its founder(s). The board must execute that mission.

Recently, new versions have been proposed in law and economics, often as a counterweight to agency theory's dominance.^17^ Also, advocates of corporate ‘purpose’, can – even if they don't invoke real entity theory explicitly – be interpreted as following a real entity paradigm. They argue that corporations should not focus on maximizing shareholder value, but on solving social problems. A specific purpose must be laid down in the articles of incorporation to function as a guideline for boards. Profits are only means to fulfilling this purpose (Mayer, 2018, pp. 7–12).^18^

In political philosophy, Abraham Singer developed a new version of real entity theory, which he calls ‘relational entity theory’. Inspired by relational views of law, Singer argues a firm is held together as a web of relational contracts. These are differentiated from spot‐contracts in standard economic markets in that persons invest themselves in the relations formed by these contracts. In a firm, cooperative norms and personal values thus play an important role to make longer‐term forms of cooperation work. This is, in Singer's view, an important source of the comparative efficiency of firms, compared to discrete market transactions. Ontologically, these relational qualities, together with group‐made decision‐procedures, rules and resources, help to explain how group cooperation can emerge, without necessarily being incorporated. Hence without relying on organicist metaphors (as Gierke did), Singer argues that a real, namely relational entity, can precede incorporation (Singer, 2018a, pp. 172–74). Corporate law comes in because the corporate legal form provides a vehicle within which firms, now understood as a nexus of *relational* forms of contracting, can flourish.

At the normative level, this also provides us with a particular interpretation of corporate purpose. As Singer states: ‘Corporations are certainly created by law and have a social function – namely to increase economic efficiency.’ (Singer, 2018a, p. 172). These efficiency considerations however on his view need to be balanced by social constraints (Singer, 2019) and ethical obligations to correct for both market and justice failures (Singer, 2018b). Hence on Singer's theory, a more complex picture emerges, at least for *business* corporations, given the particular market context in which they operate. His view reconciles real entity's traditional focus on the incorporator's freedom (the corporate purpose is whatever the group's aim is in cooperating) with the particular normative pressures that arise from operating in a market economy and democratic society.

## 5 CORPORATE GOVERNANCE: HOW IS CONTROL ORGANIZED?

Corporations' daily decision‐making is allocated to a board of directors. Boards are controlled by one or more constituencies with control rights (including information, participation, deliberation and voting rights). This raises two corporate governance questions: First, what kind of relation between boards and controlling constituencies leads to optimal fulfillment of the corporate purpose? Second, which constituency or constituencies should have control rights? The three theories of the corporation often tend to favor certain answers to these questions, but also leave space for considerable variety. The ontological and normative dimensions addressed so far guide but do often not determine the answers on the corporate governance dimension.

### 5.1 Keeping Boards to the Corporate Purpose

The first question is familiar in political theory from debates about the nature of political mandates. Should elected representatives be independent from their constituencies, or be tightly controlled by them at every step during their term? A similar basic dichotomy we find in corporate governance discussions.

Agency theory emphasizes the problem of opportunism. Agents (directors/management) may be tempted to put their personal interests first, if they are not adequately incentivized to follow the interests of their principals (shareholders). Principals have a much weaker information position and can only monitor their agents to a very limited extent. Agency theory proposes corporate governance rules to prevent a divergence of interests, and effectively bind management to shareholders' interests. First, a remuneration structure in which managerial compensation is tied to stock prices. Second, flexible takeover rules, so that the market for corporate control ensures that management will be disciplined by the threat of a takeover. Finally, shareholders can discipline managers by threatening to fire them. All of these are strategies to ensure managers are effectively incentivized to realize the expressed will of their principals.

Real entity theory tends to take an opposite view. As to the allocation of responsibility, Singer argues that ‘the board and executives owe their obligation to the corporation itself, as a whole’ (Singer, 2018a, p. 179), not to any particular constituency, such as shareholder or other stakeholders. For this he explicitly relies on the ‘team production theory’ of legal scholars Margaret Blair and Lynn Stout. They see corporations as a collaboration of team members in a productive venture (Blair & Stout, 1999).^19^ How should they organize their cooperation? The problem, as Blair and Stout see it, lies in the distribution of the surplus generated if a firm does well. If team members agree upon a fixed distribution in advance, some of them will try to shirk. But if they agree to distribute the surplus afterwards, protracted negotiations conflicts will ensue. In both cases, team members are vulnerable to each other's opportunistic behavior. The solution is the establishment of a ‘mediating hierarch’, the board. It is empowered to decide how the surplus should be distributed so that the interests of all team members are represented in a balanced way. Its independence is supposed to ensure that its authority is credible. The board serves the corporation alone.

Summing up, the potential advantage of board independence, stressed by real entity theory, is to enable a balancing of interests which keeps the team together. But against such a view, agency theorists have argued that independence is likely to give boards too many opportunities for exploiting the corporation in their own interests. Boards should be monitored more explicitly by a constituency, typically shareholders.

Concession theory does not take an explicit stance on this issue. To the extent that concession theory is associated with stronger state regulation and monitoring, one could make an argument that concession theory here aligns with agency's theory's emphasis on tight control by a principal (for concession theory: the state). The general model is integration of the corporation into a structure of public control (McMahon, 2012, pp. 82–85), but what this could mean in today's world, is very much an open question. The old practice of visitation of corporations by the King would be a very clear example of such state control (Ciepley, 2019a, p. 1005), but has been largely forgotten. Perhaps a contemporary example in the spirit of concession theory would be US Senator Elizabeth Warren's proposal to require incorporation at the federal level, impose certain public interest conditions and revoke corporate charters in case of non‐compliance.^20^

### 5.2 Choosing the Controlling Constituencies

The question who should possess control rights is also familiar from general political theory, with its debates about the boundaries of the polity and the enfranchisement of new groups (e.g refugees or children). For corporations, we can imagine a single constituency (usually shareholders) or a broader range of corporate constituencies (also workers, and/or stakeholders) to be empowered to control corporate boards.^21^

Contractualist theories land on recognizing only shareholders as corporate controllers, when they rely on the analysis of agency costs described in Section 3. However, it is important to recognize contractualist theory is not by definition committed to singling out shareholders. One can argue, based on economic theories of the firm, that other stakeholders in the firm, such as workers, should also be seen as residual claimants deserving controlling rights (Hayden & Bodie, 2020, pp. 96–102). Also, shareholder‐ownership by workers or consumers (instead of separate investors) may be the most efficient arrangement for at least some types of corporations. If so, then the landscape of optimal control structures is more varied (Hansmann, 1996).^22^ These examples show that the identification of shareholders as the only contract partners is not dictated by contractualism's most basic ontological commitments, even if most adherents in practice point to shareholders as the favored constituency.

Real entity theory, where it relies on a description of the firm as team production (see Section 4), tends to see this team as consisting of various parties: workers, investors, suppliers, customers, etc. However, we also saw that the theory stresses board independence (Section 5.1). As an ideal type, an independent corporate board would be self‐appointing (cooptation), not elected by shareholders (Kraakman et al., 2017, p. 64). This is not the case in most jurisdictions, where the standard practice is shareholder control.^23^ This practice is often accepted by real entity theories. For example, when it comes to designating a controlling constituency, Blair and Stout refer to shareholder control as the best option, following agency theory's arguments (Blair & Stout, 1999, p. 313). Singer however, is very much interested in schemes of workplace democracy, in particular in creating incentives for firms to incorporate as worker cooperatives (Singer, 2018a, pp. 190–217). Of the three theories, real entity theory as a model seems least committed to a definite position on the issue of corporate constituencies.

Finally, concession theorists are generally highly critical of shareholder control. They argue the public interest is undermined when corporate law assigns control to shareholders. Their control rights allow them to push corporate policy to the extraction of rents from third parties, i.e. the other corporate constituents or stakeholders (like workers, consumers, or taxpayers), without being liable for the risks (Ciepley, 2013, pp. 147–49).^24^ At the same time this critique doesn't lead to one clear alternative control structure, to ensure corporations do not dominate these other constituents (Claassen, 2023). Except for the special case of state‐owned corporations, control rights within the corporation hence remain private. To go beyond shareholder control, Ciepley argued that schemes of co‐determination rights for workers or ownership by industrial foundations, could both be entertained as delivering better results from a pubic interest perspective (Ciepley, 2019a, pp. 1015–18).

In different ways and to different degrees, then, all three theories of the corporation can be aligned with *other* theories, which argue for opening up corporate control rights to a wider constituency basis. Two of such other theories are workplace democracy and stakeholder theory. These theories do not provide a separate ontological interpretation of the corporation, but they can often be aligned to each of the three theories of the corporation.^25^

The most prominent constituency to gain control rights, next to shareholders, are workers. Recently, theories of workplace democracy have regained attention in political theory as an important avenue for reforming business.^26^ John Stuart Mill advocated workplace democracy as early as the nineteenth century, as an important means of teaching citizens the virtues of citizenship (Mill, 2006, p. 775). One contemporary example is Isabelle Ferreras' theory. On the analogy with many parliamentary systems, she advocates the creation of a Labor Investors' Chamber of Representatives, next to the Capital Investors' Chamber of Representatives (shareholder meeting). Any proposal from the executive board (which would operate as a government) needs to gain a majority in both chambers (Ferreras, 2017, p. 141).

A broader net is cast by stakeholder theories. In recent decades, stakeholder theories have emerged in a variety of disciplines. In management literature promoting stakeholder interests is often seen as a strategic interest; it is good for the long‐term well‐being of organizations (Freeman et al., 2010). In business ethics, the advancement of stakeholder interests is a moral obligation for boards (Donaldson & Preston, 1995; Goodpaster, 1991).^27^ In both cases, informal consultation of stakeholders can be useful. A further step would be to give stakeholders control rights, on the analogy with shareholders. Stakeholders could be added to the shareholders' meeting, so that it would effectively become a stakeholders' meeting. In dual‐board jurisdictions, representatives of stakeholders could be added to the Supervisory Board when it chooses the Executive Board, or separate Stakeholder Boards could be introduced (Deakin et al., 2018, p. 246). A key question for these stakeholder theories is *who* are and who are not included in the stakeholder basis. This depends very much on the theories proposed.^28^

The emergence of stakeholder theories led to a shareholder‐stakeholder debate (Keay, 2008; Stout, 2002). Agency theorists often raise a worry similar in spirit to their argument against board independence. Boards, when formally accountable to many or all stakeholders, cannot be effectively held accountable when their task is to balance all their interests (Heath, 2014, p. 81). When criticized by one stakeholder group, they can always hide behind the necessity of a compromise with other interests. Only when a corporation has one clear objective, such as maximizing shareholder value, is there an unambiguous way to check board performance (Jensen, 2002). Supporters of stakeholder theory have responded that there are advantages to decision‐making processes which include the voice of on the consent of all stakeholders. This forces different stakeholder groups to work together. Where they disagree, “quality conflict” arises between equally responsible parties, instead of conflicts expressed in one‐sided negative reactions (strikes, refusals to work, stress and absenteeism) from the powerless party (Ferreras, 2017, pp. 133–37).

## 6 CONCLUSION

The three theories of the corporation provide different ideal‐type ways of thinking about the ontology of corporations and the legitimacy of corporate power. The debate is multifaceted, and brings together arguments and theories from politics, philosophy, economics, law and the social sciences. Mixtures between these theories are possible, as well as concrete proposals for corporate governance reform that draw on several theories. The economics‐based version of contractualism, agency theory, has been dominant in academia for several decades. Today, the larger theoretical field is discussed again and renewed. Hopefully this paper provides stimulus to further these debates.

## ENDNOTES

^1^
For empirical literature on corporate power, see in sociology, (Crouch, 2011), in international political economy (Mikler, 2018); in political science (Van Apeldoorn & De Graaf, 2017). On corporate cultural power, see Herzog (2020).

^2^
An early call for such a political turn was Heath et al. (2010).

^3^
For brief histories of the corporation, see Mayer (2018, pp. 73–83) and Davoudi et al. (2018).

^4^
For a short survey see Kraakman et al. (2017, pp. 5–16). More detail in Blair (2003) and Hansmann et al. (2005). A critical view in Robé (2011).

^5^
‘Firms’ is the general term for economic organizations producing goods and services. Unincorporated firms are mostly sole proprietorships, or partnerships, run by a few partners who jointly pool their capital.

^6^
See the illuminating illustrations of the importance of the legal structure of the corporation, guided by Lehman Brother's casus, in Pistor (2019, p. 47 e.v.).

^7^
For overviews, see Avi‐Yonah (2005), Harris (2006), Millon (1990) and Ripken (2019). Until the 1930s, the threefold division was generally accepted in the Anglo‐Saxon legal world. Due to criticism of legal realists, it fell in disuse (Dewey, 1929). Since the 1980s, in reaction to agency theory, there is a renewed legal attention to the theories, even though the triad continues to have its critics.

^8^
For general discussion, see Bottomley (2007, pp. 40–44) and Parkinson (1993, pp. 25–32).

^9^
Thomas Hobbes's theory is a good example. See chapter XXII of *Leviation*, (Hobbes, 1991). For discussion see Claassen (2021).

^10^
In political theory see Ciepley (2013, 2019a, 2019b) and McMahon (2012). In legal scholarship, see Greenwood (2013, 2017), Padfield (2014) and Watson (2019). A popularized version is Bakan (2004).

^11^
This seems the position of some contractualists, such as Hansmann et al. (2005).

^12^
Singer develops this into a criticism of governmental provenance (Singer, 2019).

^13^
The term ‘share’ is misleading, suggesting corporations can be split into pieces like a pie and distributed among shareholders (this would violate ‘asset lock‐in’, see the introduction).

^14^
Some critics argue agency theory *fosters* business strategies that deliberately produce externalities. Limited liability incentivises shareholders to make corporations pursue risky investments, since they reap the benefits, while the costs are passed on to third parties (Mayer, 2013). When control over corporations lies with shareholders, they can push through these cost‐externalizing strategies (Bakan, 2004; Ciepley, 2019b; Robé, 2011, pp. 63–65). In terms of the residual claim assumption, others constituents are also vulnerable to the varying fortunes of corporate performance (Stout, 2002, p. 1194).

^15^
Real entity theory is connected to the philosophy of ‘pluralism’. (Runciman, 1997, xii). For the road from Gierke's Medieval corporations to business corporations, see Harris (2006).

^16^
For an analysis of the separate legal entity as a refied construct see Veldman (2018).

^17^
In law, see Blair and Stout (1999), Orts (2013), Keay (2008) and Micheler (2021); in economics (Gindis, 2009).

^18^
The real entity character of the ‘purpose’ movement emerges particularly clearly in the analysis of Levillain and Segrestin (2019).

^19^
They do not mention the historical real entity theory but do stress the corporation's nature as a ‘separate legal entity’.

^20^

https://www.warren.senate.gov/download/accountable‐capitalism‐act‐one‐pager.

^21^
A third option is to diminish corporate power itself, mainly through anti‐trust. For a trilemma around these options, see Bennett and Claassen (2022).

^22^
Still, Hansmann argued, for most firms shareholder‐ownership remains the most efficient arrangement.

^23^
However, legal theorists often point out that, in many respects, corporate boards still have a very strong autonomous position. Their fiduciary duty is towards the corporation as a whole; and also the business judgment rule provides for an important measure of independence from shareholders in case of disputes.

^24^
Ciepley argues that shareholders are neither owners nor members of the corporation. Instead, business corporations are best understood as ‘property corporations’ (*universitas rerum*), incorporations of capital. (Ciepley, 2020). On the puzzle of corporate ownership, see also Strudler (2017).

^25^
For example, stakeholder theory can be constructed as a democratized version of real entity theory, (boards become agents of all stakeholders); as extension of agency theory, giving principal status to stakehodlers (Hill & Jones, 1992); or even as a concession theory with public interest interpreted as the sum of stakeholder interests.

^26^
For an overview of the debate, see Frega et al. (2019). Classics are Dahl (1985) and Pateman (1970). Important recent contributions are Ferreras (2017), Landemore and Ferreras (2016), Anderson (2017) and Malleson (2014). For worker control from the legal/corporate governance angle, see Hayden and Bodie (2020) and Veldman (2019).

^27^
In business ethics, see also the ‘political CSR’ literature, which focuses on the external political dimension of corporate behavior. See Hussain and Moriarty (2016) and Scherer and Palazzo (2007). Corporate governance is addressed in Scherer et al. (2012).

^28^
Several models can be used to back up stakeholder theory (Boeger, 2018). E.g. see Deakin's theory of corporations as “commons” (Deakin, 2012, pp. 367, 373; see also Hiller & Shackelford, 2018).
